# Mentalizing imagery therapy for depressed family dementia caregivers: Feasibility, clinical outcomes and brain connectivity changes

**DOI:** 10.1016/j.jadr.2021.100155

**Published:** 2021-05-29

**Authors:** Felipe A. Jain, Sergey Chernyak, Lisa Nickerson, Michelle Abrams, Marco Iacoboni, Leonardo Christov-Moore, Colm G. Connolly, Lauren B. Fisher, Hitoshi Sakurai, Kate Bentley, Emily Tan, Michael Pittman, Helen Lavretsky, Andrew F. Leuchter

**Affiliations:** a Depression Clinical and Research Program, Department of Psychiatry, Massachusetts General Hospital, Harvard Medical School, Boston, MA, United States; b Applied Neuroimaging Statistics Laboratory, McLean Hospital, Harvard Medical School, Belmont, MA, United States; c Semel Institute for Neuroscience and Human Behavior, University of California, Los Angeles, CA, United States; d Department of Biomedical Sciences, College of Medicine, Florida State University, Tallahassee, FL, United States

**Keywords:** Mindfulness, Dementia, Family caregivers, Depression, Neuroimaging

## Abstract

**Background:**

Family dementia caregivers experience high rates of depression and anxiety that often go untreated due to time demands. We aimed to determine the feasibility of a brief, 4-week Mentalizing Imagery Therapy intervention, which couples mindfulness with guided imagery practices aimed at bolstering mentalizing capacity, to reduce caregiver psychological symptoms and to explore potential impact on dorsolateral prefrontal cortex connectivity.

**Methods:**

Twenty-four family dementia caregivers with moderate depression symptoms (a score of 10 in Patient Health Questionnaire-9) were assigned to either group Mentalizing Imagery Therapy (MIT, *n* = 12) or a waitlist augmented by optional relaxation exercises (*n* = 12). Participants completed questionnaires to measure depression and anxiety at baseline and followup, and those eligible also underwent resting state functional magnetic resonance (fMRI) brain imaging at these time points.

**Results:**

Eleven of 12 caregivers assigned to MIT completed the intervention and attended weekly groups 98% of the time. MIT home practice logs indicated average practice of 5 ± 2 sessions per week for 23 ± 8 min per session. All participants in waitlist completed the post-assessment. MIT participants exhibited significantly greater improvement than waitlist on self-reported depression and anxiety symptoms (*p*<.05) after 4 weeks. Neuroimaging results revealed increased dorsolateral prefrontal cortex connectivity with a putative emotion regulation network in the MIT group (*p* = .05) but not in waitlist (*p* = 1.0).

**Limitations:**

Sample size limitations necessitate validation of findings in larger, randomized controlled trials.

**Conclusions:**

A 4-week group MIT program was feasible for caregivers, with high levels of participation in weekly group meetings and home practice exercises.

## 1. Introduction

Over 15 million people in the US provide informal care for a relative with dementia (Alzheimer’s Association, 2018). Such caregiving is often stressful and has been associated with psychological consequences such as increased depression and anxiety, and physical complications including reduced immunity and early mortality. Due to time constraints, caregivers often neglect their own mental and physical care, including medical appointments. Short interventions are thought to be more feasible for this population. Mentalizing underpins our ability to understand the links between mental states and behavior in the self and others. Therapies targeting mentalizing might help caregivers improve coping by increasing interpersonal effectiveness (Jain and Fonagy, 2020). Mindfulness, or the capacity to non-judgmentally observe experience, may reduce emotional arousal and facilitate mentalizing (Allen, 2008). Therapies for caregivers that include mindfulness training, generally seven to eight weeks in length, have shown benefit for reducing depression and anxiety symptoms (Liu et al., 2017).

We tested the feasibility and efficacy of a brief, four-week “second-generation ” mindfulness therapy (Van Gordon and Shonin, 2020), Mentalizing Imagery Therapy (MIT), for family dementia caregivers with clinically relevant symptoms of depression. MIT seeks to mindfully balance self and other during mentalizing, increase participants’ understanding of the relationship between internal states and external behavior, and foster feelings of ecological connectedness (Jain and Fonagy, 2020). We hypothesized that MIT would be feasible and effective, as evidenced by high attendance and completion of home practice exercises, and that individuals receiving the intervention would demonstrate greater reduction in symptoms of depression and anxiety compared to controls. To our knowledge, the functional brain connectivity changes associated with treatment of dementia caregivers have never been explored. Because both mentalizing and mindfulness require regulation of emotions using cognitive control strategies, we also sought to identify whether brain connectivity changes between dorsolateral prefrontal cortex (DLPFC; involved in attention and executive function) and a putative emotion regulation network (with nodes in ventromedial cortex, ventrolateral prefrontal cortex and cognitive-affective cerebellum), would be observed with MIT.

## 2. Methods

Family dementia caregivers were assigned to a 4-week MIT group program or a waitlist control group during which they were provided with an audio compact disk (CD) containing progressive muscle relaxation instructions for optional self-directed use (ClinicalTrials.gov/#NCT02122068). Assignment was made according to a randomization table that had to be modified to account for magnetic resonance imaging (MRI) scanner availability. Participants in both groups who were receiving antidepressant medication or psychotherapy were advised not to change their regimen from at least six weeks prior to beginning the study through the post-study assessment. Participants were recruited from flyers and presentations at local caregiver support groups, and from among caregivers who had previously provided consent to be contacted for research at the University of California, Los Angeles (UCLA). All procedures were approved by the UCLA Institutional Review Board (protocol #13–001,877).

## 3. Participants

All participants met the following inclusion criteria for the study: Patient Health Questionnaire-9 of 10 or above (at least moderate depression symptoms) (Kroenke et al., 2001), over the age of 35 years, primary family caregiver for a relative or common law spouse of a patient with Alzheimer’s Disease or Alzheimer’s Disease Related Dementias (AD/ADRD), in contact with the individual with dementia at least three times per week for no less than 1 year, and written and oral English language fluency. Participants were excluded who had primary psychiatric diagnoses other than unipolar major depression, active substance abuse, medical instability, or history of neurological disorder, or who regularly (>2 times per week) practiced meditation or guided imagery. Participants receiving MRI additionally had no contraindications to the MR environment. Of 26 caregivers consenting to participate, 24 completed baseline assessments and are reported here (refer to supplemental CONSORT diagram).

### 3.1. Procedures

Participants underwent phone screening by a trained project coordinator, who then obtained written informed consent obtained and administered the MINI International Neuropsychiatric Inventory (Sheehan et al., 1998) and other measures. Measures were obtained at baseline and following completion of MIT or waitlist. Due to contraindications to the MR environment (such as orthopedic implants and claustrophobia), pre and post MRI scans were obtained on a subset of 8 participants in MIT and 8 in waitlist. MIT groups contained 4 to 6 participants based on recruitment flow. Home practice mindfulness and guided imagery exercises were assigned and compliance assessed with home meditation practice journals.

### 3.2. Measures

The following measures were administered at baseline and follow-up (4 weeks): 17-item clinician administered Hamilton Depression Rating Scale (HAMD) (Hamilton, 1960), 16-item Quick Inventory of Depression Symptomatology Self Report (QIDS-SR) (Rush et al., 2003), State Trait Anxiety Inventory (STAI) (Spielberger et al., 1970), Five Factor Mindfulness Questionnaire (FFMQ) (Baer et al., 2008), and the Caregiver Burden Scale (CBS) (O’Rourke and Tuokko, 2003).

### 3.3. Meditation and guided imagery training

The theoretical and practical basis of MIT has previously been described (Jain and Fonagy, 2020). Initial sessions focused on mindfully attending to internal sensations including thoughts and feelings, and later sessions focused on switching perspectives to simulate the minds of others, including in challenging situations. Training took place in weekly group meetings for 4 weeks. Manualized sessions started with gentle stretching, followed by background to understand the practices, group sharing, and practice of a different MIT technique each week (refer to Supplementary Methods). A progressive muscle relaxation CD was disseminated to those in waitlist for optional self-directed use.

### 3.4. MRI acquisition and pre-processing

Functional and anatomical T1-weighted MRI data were acquired at rest while participants fixated for 8 min on a cross and pre-processing was conducted in Analysis of Functional Neuroimages (AFNI) (refer to Supplementary Methods). Based on prior work, we adopted a rule that any scans with more than 30% of their time-series censored on the basis of motion and outlier volumes would be excluded. This led to exclusion of 3 participants from waitlist but none from MIT. We conducted a secondary sensitivity analysis including these participants’ uncensored data.

Dual regression was performed in a two-step process as previously described (Nickerson et al., 2017). Mean connectivity values for a bilateral DLPFC region of interest (ROI) were extracted from the participant-specific connectivity spatial maps. The bilateral DLPFC ROI was defined using FSL’s Talairach Atlas region corresponding to Brodmann Area 9 (spanning precentral and middle frontal gyri).

### 3.5. Statistical analysis

All statistical analysis was conducted in R. Mean interpolation was performed on missing responses for questionnaires with ≤ 2 missed responses (< 5% of questionnaires), whereas questionnaires with more than two missed responses were excluded from analysis. The data were examined for normality and square root transform applied to the HAMD prior to analysis due to leftward skew. Group differences in demographic variables were examined with unpaired t-tests or Fisher Exact tests as appropriate. Group X Time analyses were performed using linear mixed models in R, with participant as a random factor. Separate linear mixed models assessed the effects of time, adjusting for group assignment. The residuals from all linear models were examined for approximation to normality. Neuroimaging pre to post data were examined using non-parametric t-tests: paired for within group comparisons and unpaired for between group. Effect size (Cohen’s d) for change in measures was calculated as mean of the change divided by standard deviation of the change. Data are presented in the text and tables as mean ± standard deviation.

## 4. Results

### 4.1. Participants

Participants were 60 ± 10 years of age, largely female and college educated, with a high proportion suffering from major depression (Supplementary Table A). Minorities comprised about 30% of the participants overall. There were no clinically or statistically significant differences between the groups.

### 4.2. Group completion and home practice

Eleven of 12 participants completed the MIT group, and all 12 participants completed waitlist. One participant in MIT whose care recipient died during the trial was excluded from post assessments. MIT participants regularly completed home practice logs, and on average practiced 5 ± 2 times per week for 23 ± 8 min per session.

### 4.3. Clinical results

Group X Time analyses favored MIT in the QIDS-SR (*p* = .01, *d* = −1.1) and state anxiety (*p* = .02, *d* = −1.0) with large effect sizes, and a trend in the HAMD (*p* = .1, *d* = −0.7) (Table 1). There were no Group X Time effects in the FFMQ or CBS. Significant time effects suggested salutary effects of being enrolled in either study arm in the FFMQ (*p* = .05) and CBS (*p* = .1).

### 4.4. Neuroimaging results

**Fig. 1.**
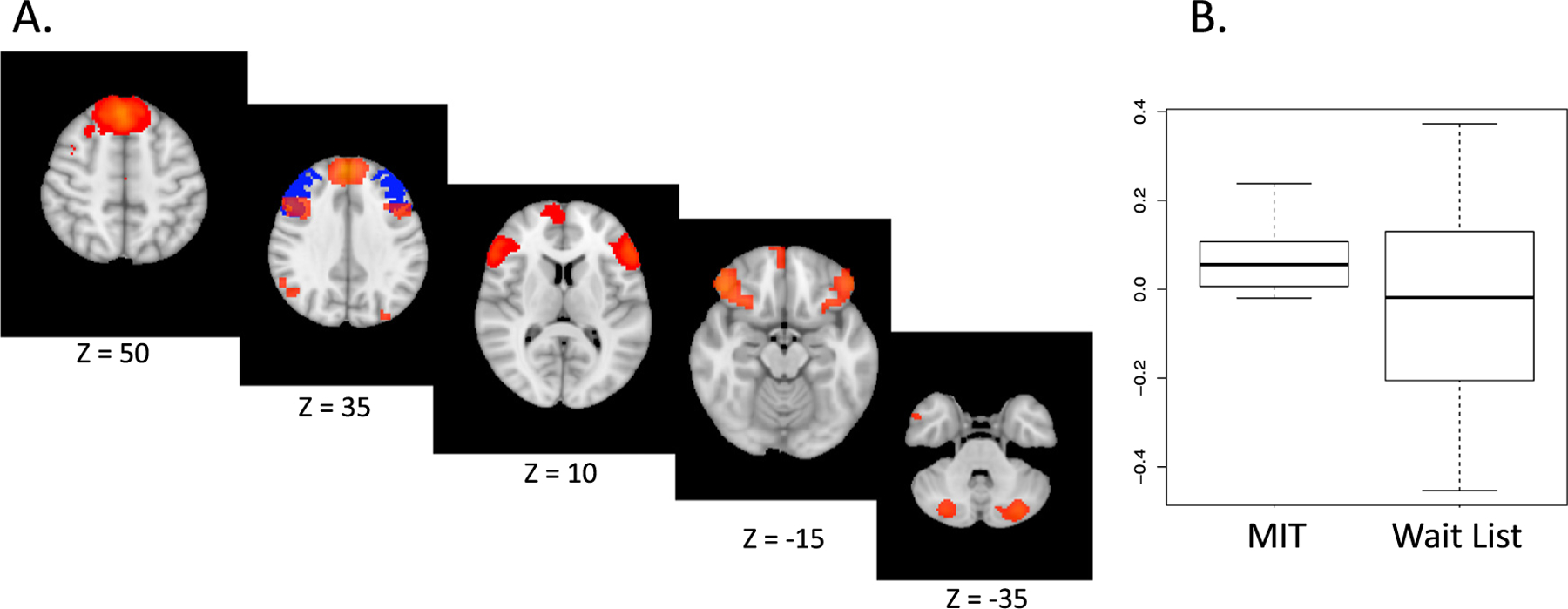
Neural connectivity change in MIT versus wait list A. Emotion regulation network shown in orange. Bilateral dorsolateral prefrontal cortex (DLPFC) region of interest in blue. B. Change in connectivity between DLPFC and network pre to post Mentalizing Imagery Therapy (MIT) (*p* = .05) or wait list (*p* = 1.0).

DLPFC connectivity with a putative emotion control network (Fig. 1 A) was assessed. There were no baseline differences between groups (*n* = 13, *p* = .4). Connectivity increased from baseline to post group in MIT (*n* = 8, *d* = 0.8, *p* = .05) but not in waitlist (*n* = 5, *d* = −0.2, *p* = 1.0) (Fig. 1 B). There was a large effect size but no significant difference between groups in connectivity change (*n* = 13, *d* = 1.0, *p* = .3). Secondary analysis including the 3 control participants initially excluded did not change these results (baseline between group differences: *n* = 16, *p* = .7; pre to post connectivity changes in waitlist: *n* = 8, *d* = −0.1, *p* = .7; between group connectivity changes: *n* = 16, *d* = 0.6, *p* = .2).

## 5. Discussion

Our results suggest that brief MIT is a feasible intervention for family dementia caregivers, who often find it difficult to participate in health promoting interventions due to time constraints. Caregivers almost invariably attended 4-week sessions and engaged with home mindfulness and guided imagery practice with a high level of frequency, on average five days out of the week. Several authors have noted barriers to recruitment of minority participants in trials of mindfulness research (Waldron et al., 2018) including with caregivers (Whitebird et al., 2013). We are encouraged that the demographics of our study indicated feasibility of participation for Black and Latinx minorities, who comprised about 30% of the overall sample. The reasons for representative rates of Black and Latinx minority inclusion are unclear but could be related to preference for a shorter intervention than typically studied, and this should be a topic for future investigation. If our results are confirmed, the 4-week MIT approach might also have practical benefits related to cost savings for therapist time that could improve scalability and dissemination relative to other stress reduction and mindfulness approaches studied for caregivers, which typically require at least 8 weeks of therapy (Lee et al., 2019).

The findings suggest favorable effects of MIT on depression and anxiety symptoms relative to waitlist control with large magnitude. However, whether accrued benefits are directly related to specific MIT effects or non-specific effects of group engagement cannot be determined, as the waitlist condition did not include group meetings. Although a time effect across groups on increasing mindfulness was found, a hypothesized superior effect of MIT was not observed, perhaps indicating that provision of relaxation exercises to waitlist participants also had salutary benefit. Limitations of the small sample size on statistical power, as well as evidence for interindividual variability of effect, indicate a need for follow up research with larger samples,

To our knowledge, this is the first clinical intervention study in family dementia caregivers to report functional brain connectivity changes resulting from intervention. Increased DLPFC network connectivity in the MIT group, but not in the control, provides one possible specific mechanism for MIT effects. 8-week mindfulness training may increase DLPFC connectivity to cingulate cortex in healthy samples (Kral et al., 2019). Our findings extend the literature by suggesting that DLPFC connectivity with a putative emotional regulation network may be increased by a shorter course of mindfulness and mentalizing imagery training. While between group statistical significance was not achieved despite a large effect size due to obvious lack of power, replication of DLPFC connectivity increase after MIT has been found in a larger trial (Jain et al., 2020), increasing confidence in this effect. We speculate that DLPFC connectivity with other network regions, such as the dorsomedial and ventromedial prefrontal cortex (known to be involved in social cognition (de la Vega et al., 2016)), and emotion regulation regions such as ventrolateral prefrontal cortex (Chiu et al., 2008; Wager et al., 2008) and cerebellum (Guell et al., 2018), might be strengthened by MIT’s focus on using interpersonal mentalizing and mindfulness strategies to promote emotion control.

We conclude that MIT is a feasible and promising intervention to alleviate the burden of depression and anxiety symptoms in family dementia caregivers, with a high level of feasibility by minority participants. Because limitations of the study design preclude attribution of symptom improvements or brain connectivity changes to specific MIT components, we suggest that future randomized, controlled trials in larger samples are necessary to secure a role for MIT in the therapeutic armamentarium.

## Supplementary Material

1

## Figures and Tables

**Table 1:** Clinical results.

	Ratings per group, No.							
	Timepoint	Wait list	MIT	Wait list	MIT	Time *p*	G* T *p*	*d*
QIDS-SR	Pre	12	12	9.1 ± 4.3	12.6 ± 6.0			
	Post	11	9	8.7 ± 5.0	5.9 ± 3.6	.02	.01	1.1
HAMD	Pre	12	12	13.2 ± 3.7	15.6 ± 5.9			
	Post	12	10	8.1 ± 4.5	7.4 ± 6.6	.0001	.1	.7
State Trait Anxiety	Pre	11	11	47.2 ± 10.8	49.2 ± 12.1			
	Post	12	9	46.1 ± 10.6	43.9 ± 9.4	.007	.02	1.0
Five Factor Mindfulness	Pre	12	12	127.4 ± 25.6	129.1 ± 23.2			
	Post	12	9	133.6 ± 28.2	131.6 ± 20.9	.05	.6	−.2
Caregiver Burden Scale	Pre	11	11	41.6 ± 18.5	51.7 ± 11.1			
	Post	11	9	39.6 ± 18.1	49.3 ± 8.5	.1	.8	.1

G*T = Group X Time; HAMD = Hamilton Depression Rating Scale 17 item; MIT = Mentalizing Imagery Therapy; QIDS-SR = Quick Inventory of Depressive Symptomatology - Self-Report. Data shown as mean ± standard deviation. Cohen’s *d* computed for between group change with positive values indicating relative benefit of MIT.
