# Supplementary material for: Mentalizing imagery therapy for depressed family dementia caregivers: Feasibility, clinical outcomes and brain connectivity changes
Source: J Affect Disord Rep. Author manuscript; Available in PMC 2021 Sep 7. (PMC8423372; doi:10.1016/j.jadr.2021.100155)
Supplement: 1 [file NIHMS1733776-supplement-1.docx]

Supplementary Methods

Mentalizing Imagery Therapy for depressed family dementia caregivers: feasibility, clinical outcomes and brain connectivity changes

Supplementary Figure 1. The Consort Flow Diagram

| Supplementary Table A. Baseline Demographic and Clinical Information | | | |
| --- | --- | --- | --- |
|  | Wait list (N = 12) | MIT (N = 12) | *p* |
| Sex (F) | 12 (100) | 10 (83) | 0.5 |
| Race |  |  | 0.2 |
| White | 9 (75) | 8 (67) |  |
| Hispanic | 0 (0) | 3 (25) |  |
| Black | 3 (25) | 1 (8) |  |
| Age | 61.6 ± 10.0 | 58.7 ± 11.4 | 0.5 |
| Years caregiving | 6.5 ± 4.0 | 5.7 ± 3.3 | 0.6 |
| Hours/week caregiving | 26.8 ± 12.6 | 28.9 ± 26.5 | 0.8 |
| Living with care recipient | 8 (67) | 7 (58) | 0.7 |
| Relation of care recipient | |  | 0.4 |
| Parent | 7 (58) | 10 (83) |  |
| Spouse | 5 (41) | 2 (17) |  |
| DSM-IV |  |  |  |
| MDD | 8 (67) | 11 (92) | 0.3 |
| GAD | 2 (17) | 7 (58) | 0.07 |
|  |  |  |  |
| Categorical data shown as number (percentage). Continuous data shown as mean ± standard deviation. F = female, GAD = generalized anxiety disorder, DSM-IV = Diagnostic and Statistical Manual IV, MIT = Mentalizing Imagery Therapy, MDD = Major Depressive Disorder. | | | |

I. Content of Mentalizing Imagery Therapy Group Sessions

1. Low-impact stretching and breath focused meditation
2. Group and partner discussion of home practice and instruction on week-varying guided imagery exercise, which have previously been described in great detail (Jain and Fonagy, 2020).
   1. Week 1: Centered Awareness (“Eye” in the Center). This practice teaches participants to localize thoughts and emotions in the body.
   2. Week 2: Nesting Doll. This exercise aims to facilitate mentalization of self at rest. The caregiver is asked to mindfully imagine a small version of themselves, including thoughts, feelings and sensations, and to bring the image within the lower chest. The caregiver is to include in the mental model their sense of breathing, their inner awareness, and their physical reality (i.e. atoms), and their spiritual beliefs about themselves if that is a component of their identity.
   3. Week 3: Situation Solver. This practice facilitates mentalization of self and the other in a challenging interpersonal situation by asking the participant to recall such a situation and imagine their mind and how their mood and emotions changed while the situation occurred. They are then asked to imagine the mind of the other person, and to imagine their interconnectedness with the other and reciprocal influences of actions and behaviors on each person’s mind. The participant is invited to situate the challenging situation within its ecological context and the time of day, and to reflect on the influences of the context on each person’s mind. Finally, the participant is asked to revision their emotional responses, actions and behaviors.
   4. Week 4: Life Globe. This practice situates the caregiver within their larger social / ecological / spiritual context as individually applicable. Participants are shown a slide show that provides images of reality at different resolutions: universe, galaxies, solar system, earth, plant and animal life, cells, atoms, and subatomic particles. The exercise then begins with mentally locating their internal center in the lower chest, and then sequentially creating mental models of self, loved ones, family or friends, communities, neighborhood, country, planet, and universe. At each stage, the participant is asked to shift perspective to imagine the mental life, or the physical qualities, of the different levels of reality.
3. Assignment of home practice exercise for upcoming week. This includes generation of a home practice plan.

II. MR acquisition and preprocessing

As previously described (Jain et al., 2019), echo-planar images (EPIs) T2∗ images were acquired on two 3T Siemens Trio Tim (Erlangen, Germany) MRI systems at the UCLA Staglin IHMRO Center for Cognitive Neuroscience or the UCLA Ahmanson-Lovelace Brain Mapping Center. Each participant’s pre and post scans were always obtained on the same scanner. A high-resolution structural T1-weighted MPRAGE scan (TE/TR = 4.9 ms/TR = 11.6 ms, flip angle = 8◦, FOV = 256 mm, slice thickness 1 mm, matrix 256 × 256 × 180) was acquired to enable functional localization and normalization to standard space. While participants fixated on a cross for 8 minutes, functional resting state data were acquired consisting of one EPI scan: Time of Echo (TE)/Time of Relaxation (TR) = 28 ms/2,000 ms, flip angle = 90◦, Field of View (FOV) = 192 mm, 64 × 64 matrix, 3 × 3 mm in-plane resolution, 34 4 mm axial slices.

Pre-processing was performed in Analysis of Functional Neuroimages (AFNI) (Cox, 1996). T1-weighted images were skull-stripped and transformed to MNI152 standard space using affine and non-linear transforms. The first three TRs of the EPI data were removed to ensure all analyzed data had reached a steady state. To minimize added smoothing, EPI data were despiked, time-shifted, motion corrected and aligned to standard space (MNI152 at 3 × 3 × 3 mm) in a single operation (Saad et al., 2009). Data were subsequently blurred with a 6 mm full width half maximum Gaussian kernel. Band pass filtering was performed at .01 to .1 thresholds. Censoring of volumes where the Euclidean norm of the six motion estimates was greater than 0.3 was performed. Outlier volumes where more than 30% of voxels were greater than the median absolute deviation of the detrended time-series were also censored. The average number of censored volumes was 17 (range 0-122).

III. Network of Interest

The network of interest was previously generated using group independent component analysis (ICA) of resting state data acquired from a larger non-overlapping sample of 30 older adults (unpublished data) using FSL Multivariate Exploratory Linear Decomposition into Independent Components Version 3.09 (Beckmann et al., 2009) with model order 45 and was selected for this study due to its prominent bilateral representation of emotion regulation regions such as dorsolateral prefrontal cortex, ventrolateral prefrontal cortex, dorsomedial prefrontal cortex, and cognitive affective cerebellum.

Supplementary Table B. Network clusters

| Cluster Index | Region | Lat | Voxels | µL | MAX X (mni) | MAX Y (mni) | MAX Z (mni) |
| --- | --- | --- | --- | --- | --- | --- | --- |
| 1 | DMPFC | B | 9182 | 73456 | 2 | 42 | 44 |
| 2 | VLPFC/FOp DLPFC | B | 2877 | 23016 | -50 | 42 | -12 |
| 3 | CB | L | 500 | 4000 | -30 | -76 | -36 |
| 4 | TPJ | R | 369 | 2952 | 44 | -62 | 28 |
| 5 | IPC | L | 293 | 2344 | -42 | -82 | 20 |
| 6 | CB | R | 289 | 2312 | 30 | -76 | -38 |
| 7 | VMPFC | R | 134 | 1072 | 2 | 54 | -18 |
| 8 | TP | R | 27 | 216 | 52 | 6 | -38 |
| 9 | SMA | R | 22 | 176 | 0 | -16 | 54 |
| 10 | IPC (PGp) | R | 18 | 144 | 48 | -76 | 18 |
| 11 | MFG | L | 15 | 120 | -40 | 24 | 42 |
| 12 | Pu | R | 12 | 96 | 29 | 63 | 31 |

aMTG = anterior middle temporal gyrus; CB = cerebellum; Cd = caudate; DMPFC = dorsomedial prefrontal cortex; FOp = frontal operculum; IPL = inferior parietal cortex; MFG = medial frontal gyrus; Pu = putamen; SMA = supplementary motor area; SPL = superior parietal lobule; TP = temporal pole; TPJ = temporopolar junction; VLPFC = ventrolateral prefrontal cortex; VMPFC = ventromedial prefrontal cortex

IV. Dual Regression

A dual regression procedure (Beckmann et al., 2009; Nickerson et al., 2017) was used to project the ICA spatial map into each participant’s resting state fMRI data to identify the subject-specific network maps and time courses. In stage 1 of the dual regression, a multivariate spatial regression of the ICA spatial map against each subject’s dataset was used to derive the subject-specific time courses (SSTC) for the ICA map. At stage 2, a multivariate regression of the SSTC against each of the subject datasets was then used to obtain the subject-specific spatial maps (SSSM) corresponding to the ICA map. The SSSM represent the connectivity map for each subject for each corresponding ICA map. Mean connectivity values for the dorsolateral prefrontal cortex region of interest were extracted from the subject specific spatial maps corresponding to the networks that were selected.

Supplementary references

Beckmann, C., Mackay, C., Filippini, N., Smith, S., 2009. Group comparison of resting-state FMRI data using multi-subject ICA and dual regression. Neuroimage 47, S148. https://doi.org/10.1016/s1053-8119(09)71511-3

Cox, R.W., 1996. AFNI: software for analysis and visualization of functional magnetic resonance neuroimages. Comput. Biomed. Res. 29, 162–173.

Jain, F.A., Connolly, C.G., Moore, L.C., Leuchter, A.F., Abrams, M., Ben-Yelles, R.W., Chang, S.E., Ramirez Gomez, L.A., Huey, N., Lavretsky, H., Iacoboni, M., 2019. Grief, Mindfulness and Neural Predictors of Improvement in Family Dementia Caregivers. Front. Hum. Neurosci. 13, 155. https://doi.org/10.3389/fnhum.2019.00155

Jain, F.A., Fonagy, P., 2020. Mentalizing Imagery Therapy: Theory and Case Series of Imagery and Mindfulness Techniques to Understand Self and Others. Mindfulness (N. Y). 11, 153–165. https://doi.org/10.1007/s12671-018-0969-1

Nickerson, L.D., Smith, S.M., Öngür, D., Beckmann, C.F., 2017. Using Dual Regression to Investigate Network Shape and Amplitude in Functional Connectivity Analyses. Front. Neurosci. 11. https://doi.org/10.3389/fnins.2017.00115

Saad, Z.S., Glen, D.R., Chen, G., Beauchamp, M.S., Desai, R., Cox, R.W., 2009. A new method for improving functional-to-structural MRI alignment using local Pearson correlation. Neuroimage 44, 839–848. https://doi.org/10.1016/j.neuroimage.2008.09.037
